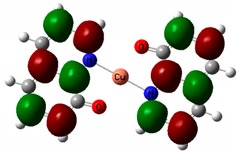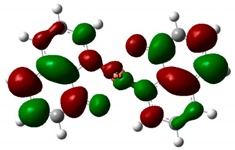# Selected Organometallic Compounds for Third Order Nonlinear Optical Application

**DOI:** 10.3390/nano9020254

**Published:** 2019-02-13

**Authors:** Anna Popczyk, Aouatif Aamoum, Anna Migalska-Zalas, Przemyslaw Płóciennik, Anna Zawadzka, Jaroslaw Mysliwiec, Bouchta Sahraoui

**Affiliations:** 1 Faculty of Chemistry, Department of Engineering and Advanced Materials Modelling, Wroclaw University of Science and Technology, Wyb. Wyspianskiego 27, 50-320 Wroclaw, Poland; 2 Laboratoire MOLTECH-Anjou, Université d’Angers, CNRS UMR 6200, 2 Boulevard Lavoisier, 49045 Angers, France; bouchta.sahraoui@univ-angers.fr; 3 Laboratory of Physics of Condensed Matter (LPMC), Faculty of Science, Chouaib Doukkali University, El Jadida 24000, Morocco; aamoumaouatif@gmail.com; 4 Department of Automation and Measurement Systems, Faculty of Physics, Astronomy and Informatics, Nicolaus Copernicus University, Grudziadzka 5, 87-100 Torun, Poland; przemas@fizyka.umk.pl; 5 Institute of Physics, Faculty of Mathematics and Natural Sciences, J. Dlugosz University of Czestochowa, Al. Armii Krajowej 13/15, 42-200 Czestochowa, Poland; amigalskazalas@gmail.com

**Keywords:** organometallic compounds, nonlinear optic (NLO), physical vapor deposition (PVD), third harmonic generation (THG), coordination complexes, DFT/B3LYP/6-31G(d) calculations

## Abstract

In this paper, we present the third harmonic generation response of Znq_2_ (Bis-(8-hydroxyquinolinato)zinc), Cuq_2_ (8-Hydroxyquinoline copper(II)), and Alq_3_ (Tris-(8-hydroxyquinoline)aluminum) organometallic compounds. An experiment was conducted for s and p polarizations of incident beam, using the Maker fringes technique. The third order nonlinear susceptibility χ^(3)^ was estimated using the Kubodera and Kobayashi comparative model, on the grounds that presented compounds exhibit high linear absorption of the generated third harmonic wavelength (355 nm). These complexes were deposited as thin films using the physical vapor deposition (PVD) method. Investigated complexes vary in terms of the coordination center and number of quinoline ligands, which visibly influence their nonlinear response. The global hybrid B3LYP functional with the basis set 6-31G(d) was used in computing the linear and non-linear optical properties. The computed γ_tot_ value (8765.36 × 10^−36^ esu for Cuq_2_) is superior to that of methylene blue (γ = 32.00 × 10^−36^ esu). The calculated theoretical values were found to be in good agreement with the experimental results.

## 1. Introduction

Nowadays, a great number of researchers pay attention to the metal complexes that represent promising candidates for nonlinear optics (NLO) [1]. These kind of compounds attracted enormous attention due to their applications in different fields, such as medicine [2], organic light-emitting diodes (OLED) [3,4], photovoltaic [5], and photonics and optoelectronics [6,7]. Organometallic compounds exhibit potential as a third order nonlinear material, due to the important charge transfer between the ligands and the metal, as well as the switchable nonlinearity that is related to multiple electronic states of the central metal atom [8,9]. It has been already proven that the abovementioned properties allow for significant values of first molecular hyperpolarizability β [10,11]. Bis(8-Hydroxyquinoline) zinc—Znq_2_, bis(8-Hydroxyquinoline) copper (II)—Cuq_2_, and tris(8-Hydroxyquinoline) aluminum—Alq_3_ have been widely investigated in recent years as organometallic compounds with potential applications in modern optoelectronics [12,13,14,15,16]. The organometallic complexes combine the advantages of organic and inorganic materials, such as an ease of fabrication, architectural flexibility, excellent temporal as well as physical ruggedness, and high resistance to laser light [17,18,19]. These kind of compounds are distinguished from others as, besides π→π* transitions present in the organic system, they can also exhibit intramolecular charge transfer (ICT) from ligand to metal (LMCT), from metal to ligand (MLCT), or d-d transitions [20]. In coordination complexes d-orbitals of transition metals have the ability to interact with the conjugated π-electron orbitals of the ligand. This phenomenon visibly enhances the possibility of a third order susceptibility (χ^(3)^) tailoring process and better design of molecules for NLO purposes [21]. The NLO-effects of the organometallic compounds originate from the interaction between an incident light and the electrons inside the individual molecular units, which allows a good nonlinear optical response to be achieved [22]. Presented compounds contain two or three organic ligands attached to the transition metal through chelation. The 8-hydroxyquinoline ligand shows electro-donating properties due to the presence of nitrogen atom at the quinoline ring and a phenolate oxygen atom [23]. Chosen metals are situated in the center of the coordination complex and exhibit differing electronegativity, which is the individual property of the metal atom, describing its tendency to attract pairs of shared electrons toward itself. The abovementioned properties suggest a significant charge transfer in these compounds, which can be induced by high-intensity light. The third harmonic generation (THG) is one of the experimental techniques from the nonlinear optics field, where the generated light has a tripled frequency of incident beam [24]. This technique, in contrast to the second harmonic generation, can be investigated for centrosymmetric molecules, as well as non-centrosymmetric. This phenomenon can be described by the third order susceptibility (χ^(3)^). It was shown in previous studies that systems with the highest charge transfer between donor and acceptor parts and long conjugation path achieve the highest values of nonlinear response [25,26]. In this paper, we present the third order nonlinear optical properties of three organometallic compounds (Znq_2_, Cuq_2_, and Alq_3_) induced by a Nd:YAG pump laser and measured for polarization s and p of the incident beam.

**Figure 1:**
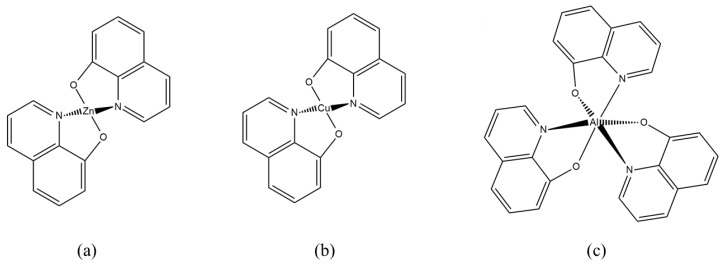
Chemical structures of investigated molecules: (**a**) 8-Hydroxyquinoline zinc [Znq_2_], (**b**) 8-Hydroxyquinoline copper II [Cuq_2_], (**c**) Tris(8-Hydroxyquinoline) aluminium [Alq_3_].

Due to the size and complexity of the structures of the analyzed molecules, semi-empirical methods such as the Lee model, Kurtz-Perry, Kobajaschi-Kubadera, and Rentjes [27,28,29,30] play an important role in the diagnostics, control, and investigations of molecular nonlinear optical properties. The model of François Kajzar is also commonly used [31]. It is worth mentioning that previously, third order nonlinear response in coordination complexes was mostly measured with the Z-Scan technique, whereas in this paper we present results from THG. Comparison and confrontation of theoretical and experimental results allow for a continuous evaluation of the quality of predictions and possible modification of the applied techniques and methods. Using quantum chemical methods, it has become possible to construct a clear description of the relationship between the electronic structure of the molecule and its nonlinear optical response. This allows for not only a deep understanding of phenomena related to the interaction of light with molecular matter, but also to organize the rich and ever-expanding experimental material in the presented area of research. In most cases, theoretical modeling is the starting point for the synthesis of functionalized molecular systems with desirable properties. Hence, many research groups were able to optimize the *β* and γ values by modifying the π-electron core, optimizing the donor (D) and acceptor (A) groups, and through conformational tuning of the studied systems [32,33,34,35]. To find a correlation between molecular structure and NLO phenomena, we extended our research to calculate second order hyperpolarizability. Figure 1 presents the chemical structures of investigated compounds.

The experimental studies used have allowed us to verify the correctness of the construction of the applied computational theoretical model, which together allowed for analysis of the mechanisms of the physical processes responsible for nonlinear effects.

## 2. Materials and Methods

### 2.1. Preparation of Thin Films

Presented compounds were obtained commercially from Sigma Aldrich Chemical Company (99% for Znq_2_, 98% for Cuq_2_, and 99.995% for Alq_3_, St. Louis, MO, USA). The Znq_2_, Cuq_2_, and Alq_3_ thin films were fabricated with the Physical Vapor Deposition (PVD) technique using Thin Film Deposition System—NANO 36™ (Kurt J. Lesker Company, Jefferson Hills, PA, USA) in a high vacuum chamber under 2 × 10^−6^ Torr. Compounds were evaporated from the ceramic effusion cell with source material and deposited successfully on BK7 glass substrates kept at room temperature during the deposition process. Thickness of the thermally evaporated films depends on properties of the source, time of evaporation, and distance between source and substrate. The powdered source material was evaporated from a ceramic effusion cell with a nozzle 10 mm in diameter on the top. The distance between source and substrate was equal to 20 cm and substrates were rotated at a speed equal to 20 rot/min during the whole deposition process. The deposition rate was considered the most important parameter for thin films forming and for all samples it was equal to 0.1 Å/s. Initial power necessary to achieve the desired rate of the source material’s evaporation depends on properties of the source material, i.e., molecular mass, density, and temperature of the sublimation. The initial power required to fix the rate at 0.1 Å/s was different for all studied complexes and was equal to 280 W (Znq_2_), 220 W (Cuq_2_), and 150 W (Alq_3_), respectively. The deposition rate and the final film’s thickness were controlled by the piezoelectric controller.

### 2.2. UV-Vis Absorption Spectra

A Lambda 950 UV/Vis/NIR spectrophotometer (Perkin Elmer, Waltham, MA, USA) was used to measure the absorption spectra of Znq_2_, Cuq_2_, and Alq_3_ thin films in the range 300–1100 nm at room temperature.

### 2.3. Atomic Force Microscopy (AFM)

An Agilent 5500 Atomic Force Microscope equipped with a MSNL-D Bruker cantilever was used to measure AFM images. AFM imaging was carried out in the tapping mode. Measurements were performed in an isolated sound and vibration proof chamber at a temperature equal to 16 °C.

### 2.4. NLO Measurements

The nonlinear optical measurements were carried out using the rotational Maker fringe technique [36,37,38,39]. The Nd:YAG laser generating at λ = 1064 nm was used as the fundamental beam, with 10 Hz repetition rate and 30 ps pulse duration. The energy of every pulse was 100 µJ and it was controlled by the laser powermeter (LabMax TOP, Coherent, Santa Clara, CA, USA). The polarizer and the half wave plate were used for polarization adjustments of the laser beam. The incident beam was focused on the sample by the lens with the focal point equal to 25 cm. The motorized rotation stage changed angle of incidence from −60° to +60°. The interference filter was used to ensure only measurement of generated third harmonic wavelength (355 nm). The signal was recorded by the Hamamatsu R1828-01 photomultiplier which collected intensity of THG every 0.5°. The boxcar average system integrated the signal which was afterwards processed by the computer. As the reference material, we used a silica glass plate (SiO_2_) with a well-established value of χ^(3)^ equal to 2.0 × 10^−22^ m^2^V^−2^. Due to the high linear absorption in the region of the generated third harmonic wavelength, the Kubodera and Kobayashi theoretical model was used to calculate the third order susceptibility of investigated compounds [40,41,42]. This model makes a comparison between the maximum light intensity of the generated signal of THG and the maximum light intensity of the reference signal (silica).

(1) $$\chi^{\left(3\right)}=\chi_{silica}^{\left(3\right)}\left(\frac{2}{\pi }\right)\left(\frac{L_{coh}}{d}\right)\left(\frac{\frac{\alpha d}{2}}{1-exp\left(-\frac{\alpha d}{2}\right)}\right)\sqrt{\frac{I_{comp}^{3\omega }}{I_{silica}^{3\omega }}}$$

where, *L_coh_* represents the coherence length of the silica equal to 6.7 µm. The thickness of the studied thin films is represented as *d*, $\chi_{silica}^{\left(3\right)}$ is the third nonlinear susceptibility of fused silica, $I_{comp}^{3\omega }$ and $I_{silica}^{3\omega }$ represent the average peak intensity of the recorded Maker fringes signal. The thicknesses of the prepared thin films were measured using the profilometer Veeco Dektak 3.

### 2.5. Computational Details

The ground-state geometries were optimized in gas phase by a density functional theory DFT/B3LYP called the hybrid method based on three Becke parameters using the base 6-31G(d) level of theories implemented in the GAUSSIAN 09 program package [43,44]. With this method the dynamic second order hyperpolarizability γ (−2ω; ω, ω, 0) and the HOMO and LUMO energy levels of the investigated molecules were calculated. The addition of d polarization functions on the carbon and nitrogen atoms are critical in order to have a precise estimation of the hyperpolarizabilities.

## 3. Results

### 3.1. Topography and Structural Properties

**Figure 2:**
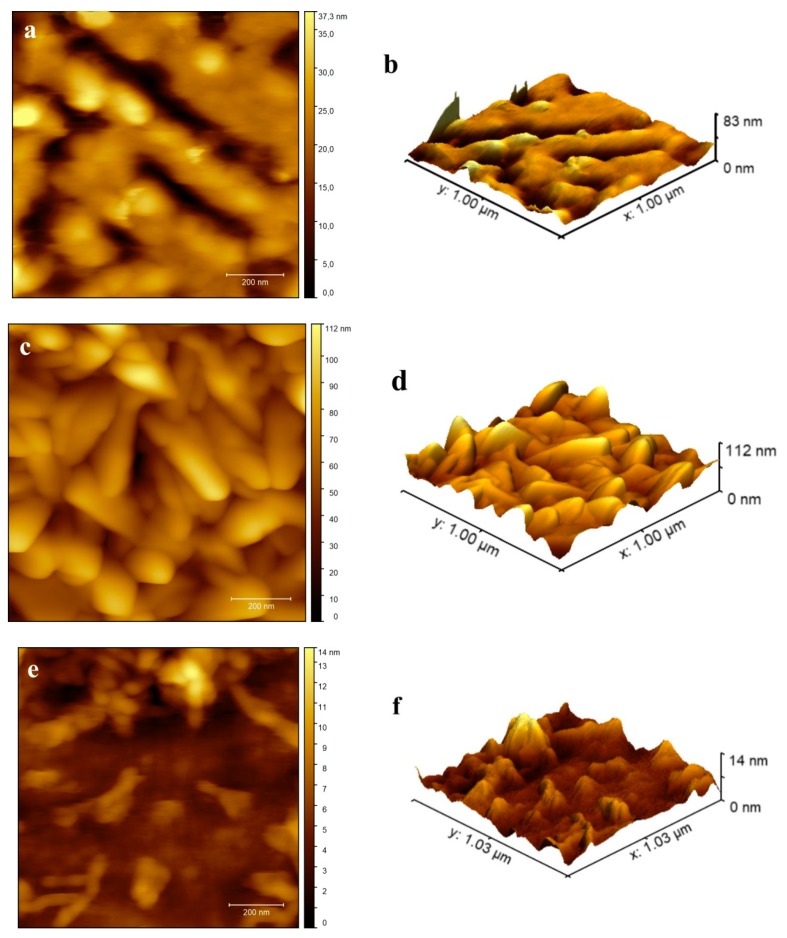
Topography Atomic Force Microscopy (AFM) images of investigated metalorganic complexes thin films: Znq_2_ (**a**) 2D and (**b**) 3D, Cuq_2_ (**c**) 2D and (**d**) 3D, and Alq_3_ (**e**) 2D and (**f**) 3D.

The surface morphology of the investigated metalorganic thin films deposited by the PVD technique was analyzed by Atomic Force Microscopy (AFM). Figure 2a–f shows AFM images of the Znq_2_, Cuq_2_, and Alq_3_ thin films for samples deposited on the substrate at room temperature, respectively. The scanning area of the films was equal to 1 × 1 µm^2^. Presented figures were drawn using Gwyddion software and they visualize 2D and 3D images for all of the investigated metalorganic complexes.

**Figure 3:**
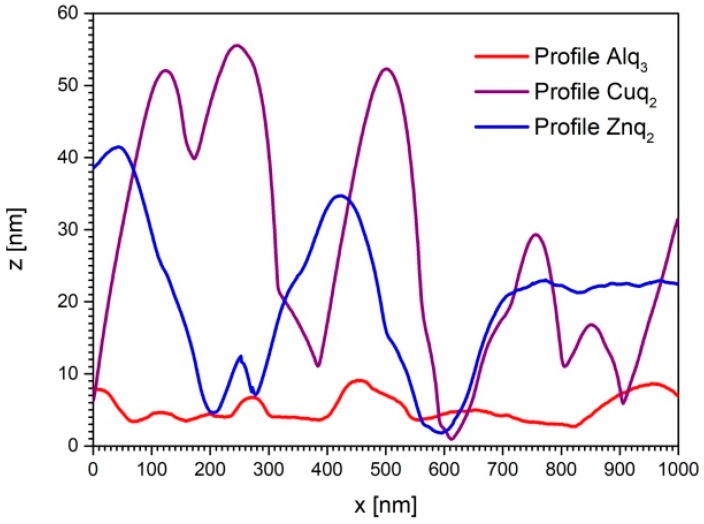
AFM profiles of investigated metalorganic complexes thin films.

The Znq_2_ and Cuq_2_ thin films deposited at room temperature showed nanocrystalline characters. The structure of the films contained clearly visible hills of nanocrystallites and valleys between them. The average differences between them were equal to ca. 40 nm for Cuq_2_ and 30 nm for Znq_2_. The structure of the Alq_3_ thin film was completely different. The structure also showed a nanocrystalline character, but the size of the nanocrystallites were much smaller and the surface was more homogeneous. The average difference was equal to ca. 4nm. Thin film profile examples of tested compounds are presented in Figure 3.

**Figure 4:**
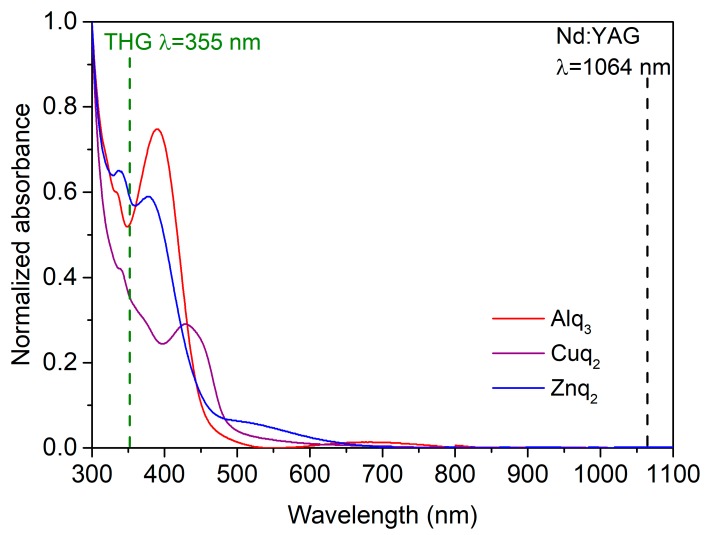
UV-Vis absorption spectra of studied molecules (Znq_2_, Cuq_2_, and Alq_3_).

**Figure 5:**
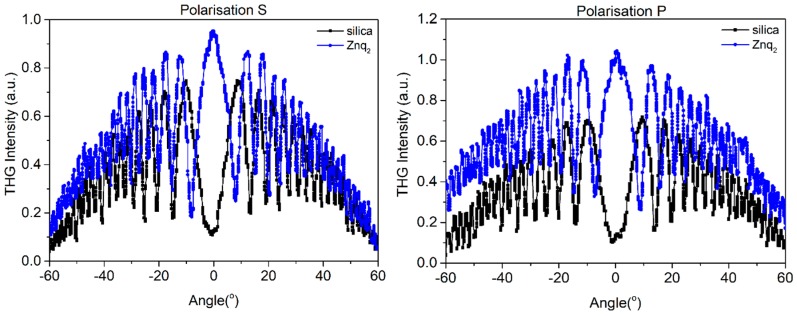
Third harmonic intensity of Znq_2_ film and silica as a function of the incident angle for polarization s and p.

AFM images showed that the different 8-Hydroxyquinoline derivatives form different nanocrystalline thin films despite the fact that the deposition process was carried out under identical vacuum conditions and deposition rate. However, replacement of the metal atom in the Mq_2_ (M = Zn, Cu) molecule hardly changes the structure of the films. Finally, both fabricated Mq_2_ thin films had a much larger active surface compared to the Alq_3_ film. Formation of the structures visible in Figure 4 and Figure 5 also indicates various mechanisms of thin film formation for Mq_2_ compared to Alq_3_ molecules. Film growth of Mq_2_ complexes showed an island formation nature. In the case of the Alq_3_ complex, film growth showed definitely more layer by layer growth. This phenomenon indicated a much stronger interaction between the molecules Mq_2_ compared to Mq_3_ on the substrate’s surface, not only during the formation process of the thin films, but also after the deposition inside thin films. Such intermolecular interactions usually affect nonlinear optical properties of the thin films. There are many interesting structural effects that can affect second and third order nonlinear effects. The enhancement of THG responses by surface plasmons [45,46], metal’s nanoparticles [47,48], or well-oriented nanostructures has been reported among many researchers [49,50]. There is still a need for systems (compounds) that can exhibit large NLO responses for potential photonics applications as nonlinear optical (NLO) systems play a major role in the field of nanophotonics, photonics including optical information processing, sensor protector applications, and optical data storage. As the occurrence of NLO response is not well studied in the case of investigated compounds, we studied these interesting NLO systems. In such systems, intermolecular interactions are not strong enough to create a well-oriented nanostructural film, which was proven by visualization of surface morphology. Moreover, the internal molecular bonds are not broken during the sublimation process. Therefore, the nanostructured nature of the films is entirely random, nanoparticles are not formed on the surface, and there are no plasmonic effects.

### 3.2. Optical Properties

Figure 4 illustrates the UV-Vis absorption spectra of Znq_2_, Cuq_2_, and Alq_3_ thin films. All of the investigated compounds exhibit absorption in range of the blue light, with maxima positioned at λ_abs_ = 380 nm for Znq_2_, λ_abs_ = 390 nm for Alq_3_, and λ_abs_ = 428 nm for Cuq_2_. These bands might be attributed to the combination of intraligand π→π^*^ transition and the ligand to metal charge transfer (LMCT). The most important observation from this measurement is the fact that none of the compounds absorb wavelengths of 1064 nm, which was used to investigate the third nonlinear optical properties (THG response). These results suggest that in case of third order nonlinear susceptibility calculations, the use of the Kubodera-Kobayashi comparative model is justified, due to the strong absorption of generated third harmonic wavelength (355 nm).

### 3.3. THG Measurements

**Figure 6:**
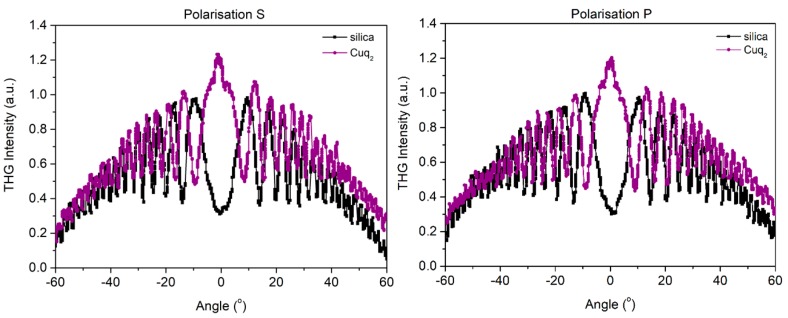
Third harmonic intensity of Cuq_2_ film and silica as a function of the incident angle for polarization s and p.

**Figure 7:**
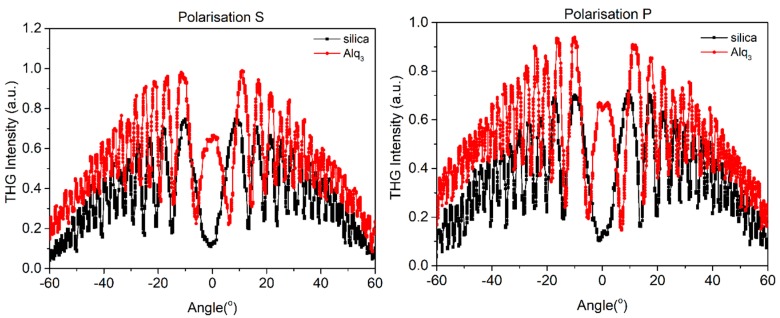
Third harmonic intensity of Alq_3_ film and silica as a function of the incident angle for polarization s and p.

In Figure 5, Figure 6 and Figure 7 we present curves with an oscillatory signal of measured sample and reference for two different polarizations of pump beam. There is a good symmetry of signals at incident angle 0° visible for all of investigated samples. In every case, there is no significant difference between applied polarizations of initial laser beam. The angle range of −60° to +60° allowed us to measure a wide spectrum with observable growth and decay of Maker fringes intensity and phase matching. All of the presented compounds exhibit a THG efficiency two magnitudes higher than reference material. Table 1 represents the thickness of studied samples, their absorption coefficients, and their third order susceptibilities, which were calculated using Equation (1).

A few important conclusions can be drawn from comparing the results of calculated third order susceptibilities. Due to the strong absorption coefficient in the region of generated third harmonic wavelength, the figure of merit was calculated (χ^(3)^/α) to allow easier comparison of NLO response for investigated compounds. This value provided a more accurate figure, which helped to conclude a clearer influence of the metal center on THG. Firstly, Alq_3_ has the lowest THG response, whereas Cuq_2_ have visibly the highest one. This order might be due to the metal’s electronegativity, orbitals’ shape, or extended delocalization path that are characteristic for coordination complexes. It should be noted that in the cases of both Cuq_2_ and Znq_2_ the clear intramolecular charge transfer is visible between ligand and metal (See Table 4), which increases THG response. Additionally, the unfilled d-shell in case of Cu enhances the NLO response, what can be associated with the additional electronic levels present in this system and increase of electron-accepting properties [51]. Moreover, in case of Alq_3_, the lowest THG response might originate from the lack of intramolecular charge transfer between ligand and metal center (See Table 4). Electron configuration of Al^3+^ indicates that all orbitals are filled with electrons, hence its acceptor properties visibly decrease. For this sample quinolone ligands seem to act as a donor and an acceptor simultaneously. In the investigated compounds of Znq_2_ and Cuq_2_, an organic ligand has more of an electron donating character, hence its orbitals have lower energy than metal orbitals that resolve in electron transfer from bonding π or antibonding π* orbital into σ* orbitals. Thus, the nature interactions between ligand and metal point to the ligand to metal charge transfer (LMCT) as an origin of high nonlinear optical response [52,53]. In the case of presented coordination complexes, the influence of the polarization of the incident beam is negligible. Results for p as well as s polarization vary in an insignificant way.

### 3.4. Theoretical Simulations

**Figure 8:**
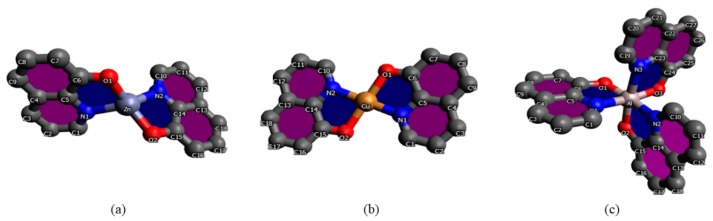
Optimized structures of investigated molecules: (**a**) Znq_2_, (**b**) Cuq_2_, (**c**) Alq_3_.

**Figure 9:**
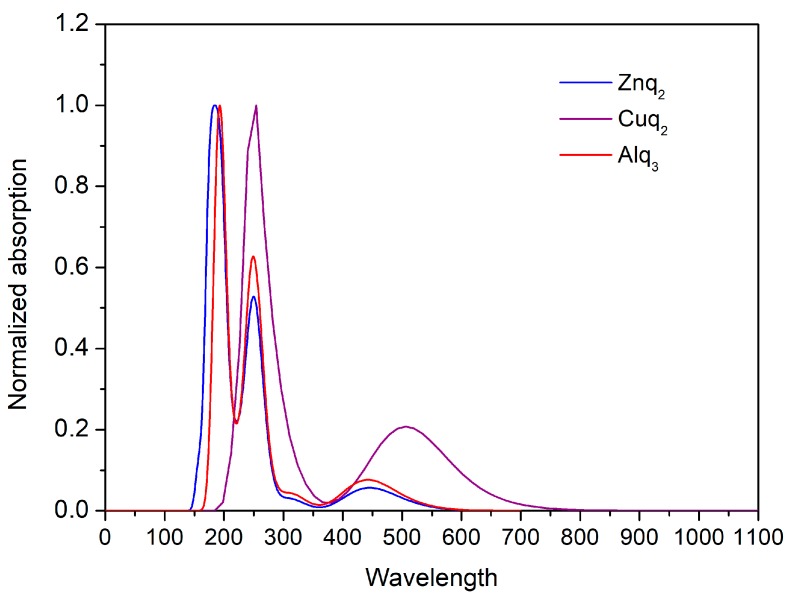
Theoretically simulated UV-Vis spectra of Alq_3_, Znq_2_, and Cuq_2_.

The optimized structures of investigated compounds using the DFT method are shown in Figure 8. The linear optical properties are very important for the characterization of materials used in nonlinear optics. Figure 9 displays the calculated UV–Vis absorption spectra of Alq_3_, Znq_2_, and Cuq_2_ complexes. The theoretical spectrum was made in the gas phase and the absorption peaks are shifted towards shorter wavelengths in respect to the experimental spectrum (see Table 2). The shift of the experimental absorption band towards the longer wavelengths is caused by the amorphous nature of the structure of films obtained by the PVD method. Comparing the experimental UV-visible absorption spectra and theoretical, it can be seen that intermolecular interactions cause a bathochromic effect on all molecules [54].

Calculated values of HOMO and LUMO energy and the HOMO-LUMO energy gap (see Table 2) allows you to explore the relationship of non-linear properties with the structure of the studied materials.

The HOMO-LUMO gap of Znq_2_ is relatively higher than the energy gap value for the remaining molecules. The high energy gap and the data here suggests that Znq_2_ is relatively less reactive than the molecules Alq_3_ and Cuq_2_. Decreasing the value of the energy gap increases the charge transfer within the particles. Persistent particles have large energy breaks, and the more reactive particles have small energy breaks HOMO-LUMO. Lower HOMO-LUMO gaps usually herald better nonlinear properties which are also visible in these complexes. It is known that the dependence of the energy gap on the third order nonlinear optical susceptibility is represented as the inverse of the optical break raised to 6th power [55].

(2) $$\chi^{\left(3\right)}=\frac{1}{{E_{g}}^{6}}$$

The total third order hyperpolarizability *γ_tot_* values have been calculated from the formula presented in Reference [56]. As you can see from Table 3, the hyperpolarizability of complex Cuq_2_ (γ_tot_ = 8765 × 10^−36^ esu) is about one order higher than for Alq_3_ (*γ_tot_* = 359 × 10^−36^ esu) and two orders higher than for Znq_2_ (*γ_tot_* = 57 × 10^−36^ esu). We compared our hyperpolarizability values with those reported by Leupacher et al. [57] of methylene blue (*γ* = 32 × 10^−36^ esu) by means of THG measurements and the value of Cuq_2_ is two orders higher. A significant value of the corresponding component points to the delocalization of the electrons in a given direction. The second order frequency-dependent hyperpolarizability for Cuq_2_ is dominated by the oblong component of γ_zzzz_. The calculated values suggest that the hyperpolarizability is controlled by donor–acceptor strengths and the metal cation variability.

As mentioned above, this complex exhibits an intramolecular charge transfer and produce polarization along the π-conjugated bonds. Table 4 shows the visualized structures of studied complexes and show the population of electrons on their orbitals. The left column shows molecular orbitals that are occupied in the ground state, with the lowest-energy orbital at the top. The orbital wave functions are positive in the red regions and negative in the green. The right column shows virtual molecular orbitals which may be occupied in excited states.

The HOMO for Tris-(8-hydroxyquinoline) aluminum—Alq_3_ is mainly located on the three quinoxaline ligands. LUMO is mainly localized on the two nitrophenyl quinoxaline ligands. For the remaining two investigated molecules, the HOMO is also located on the ligands. LUMO is located all over the whole complex alike on the two linked ligands and as well as on Zn and Cu atoms. This electron delocalization can be attributed to the strong electron withdrawing nature of this group and is defined as ligand to metal charge transfer (LMCT) leading to high stability of complex. This effect is especially seen for the Znq_2_ and Cuq_2_ compounds. The presented calculation results reproduce the trend of the experimental results well and show clearly the increase of the nonlinear optical response upon Cu metal complexation [58].

## 4. Conclusions

The Alq_3_, Znq_2_, and Cuq_2_ complexes were studied both experimentally and theoretically, the HOMO and LUMO levels of compounds were analyzed by DFT/B3LYP/6-31G(d) method. In this paper, the main aim was to compare the third harmonic generation responses of three organometallic compounds investigated in the form of thin films. The films were obtained by using the PVD technique in a high vacuum on BK7 glass substrates. Structural properties showed that the number of quinoline ligands have a significant influence on the mechanism of thin film formation as well as the final microstructure of the film. The THG measurements were performed by means of the third harmonic generation (THG) technique at the fundamental wavelength of 1064 nm. The third order susceptibilities $\chi_{THG}^{\left(3\right)}$ were calculated using the Kubodera and Kobayashi comparative model. We obtained the highest THG response for Cuq_2_. These results indicate that the character of the metal as well as the shape of its orbital strongly influence the NLO response. The thin films fabricated by the Physical Vapor Deposition technique have a high quality, give correct experimental results, and have good compliance with the obtained theoretical data. It is clearly shown that the substitution by Cu atom has affected the lowering of the HOMO-LUMO bandgap and at the same time leads to an increasing of the hyperpolarizability γ_tot_. The HOMO-LUMO map indicates that electron delocalization in the studied complexes can be attributed to ligand to ligand charge transfer (LLCT) for Alq_3_ and to ligand to metal charge transfer (LMCT) in the case of Cuq_2_ as well as Znq_2_. The obtained values of second order hyperpolarizability γ, (which characterize the individual molecule) for Cuq_2_ is much larger than the values obtained for the remaining molecules from investigated series (more than 22 times). The advantage of coordination complexes is the additional effects which are present in organometallics that result from efficient delocalization of π–electrons and influence the nonlinear optical behavior. The crucial advantage of the studied materials is the unique combination of good nonlinear optical and electronic properties and good optical quality of the studied films which are extremely difficult to be achieved. We believe that NLO properties of the presented organometallic thin films can be used in optoelectronic devices due to their high value of third harmonic generation.

## Figures and Tables

**Table 1:** The values of the third order nonlinear optical susceptibility $\chi_{THG}^{\left(3\right)}$ of thin films obtained from the third harmonic generation (THG) technique for polarization s and p.

			Polarisation S		Polarisation P		
	d [nm]	α [10^6^ m^−^^1^]	χ^(3)^ [10^−^^20^ m^2^/V^2^]	χ^(3)^ [10^−^^12^ esu]	χ^(3)^ [10^−^^20^ m^2^/V^2^]	χ^(3)^ [10^−^^12^ esu]	χ^(3)^/α [10^−^^19^ esu·m]
**Znq_2_**	173	4.19	1.62	1.16	1.76	1.27	3.03
**Cuq_2_**	166	3.65	1.85	1.32	1.78	1.27	3.62
**Alq_3_**	223	3.81	1.32	0.96	1.33	0.96	2.51
**Silica**	-	-	0.02	0.16	0.02	0.16	

d—thickness, α—absorption coefficient, χ^(3)^—third order susceptibility.

**Table 2:** HOMO and LUMO energy levels and theoretical bandgap (E_g_)_HOMO–LUMO_ (B3LYP/6-31G(d)).

Sample	HOMO [eV]	LUMO [eV]	(E_g_)_HOMO–LUMO_ [eV]
Alq_3_	−5.25	−2.19	3.06
Znq_2_	−5.42	−2.06	3.36
Cuq_2_	−5.13	−2.16	2.97

**Table 3:** Some selected components of the frequency-dependent **γ** (−2ω; ω, ω, 0) values at ω = 0.042827 a.u. = 1064 nm.

Sample	*γ_xxxx_* × 10^−^^36^ esu	*γ_yyyy_* × 10^−^^36^ esu	*γ_zzzz_* × 10^−^^36^ esu	*γ_xxyy_* × 10^−^^36^ esu	*γ_xxzz_* × 10^−^^36^ esu	*γ_yyzz_* × 10^−^^36^ esu	*γ_tot_* × 10^−^^36^ esu
**Alq_3_**	626	623	14	211	28	28	359
**Znq_2_**	213	13	6	9	9	10	57
**Cuq_2_**	1751	406	25653	222	6369	1417	8765

**Table 4:** The frontier molecular orbitals: HOMO and LUMO for Alq_3_, Znq_2_, and Cuq_2_.

Sample	HOMO	LUMO
**Alq_3_**	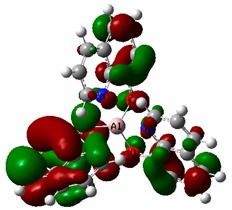	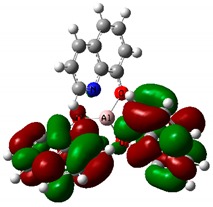
**Znq_2_**	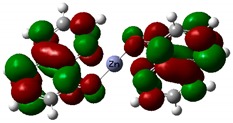	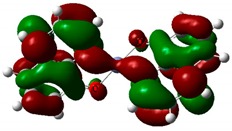
**Cuq_2_**